# Research challenges in palliative and end of life care

**DOI:** 10.1136/bmjspcare-2015-001091

**Published:** 2016-03

**Authors:** Irene J Higginson

As the number of older people increases across the world, and more people approach the end of their lives with chronic and complex conditions, the need for robust and relevant research in palliative and end of life care has never been greater. An estimated 19 million people need palliative care worldwide each year,^1^ and evidence to help provide relief from symptoms and offer support to patients and those close to them at the end of their lives is an ongoing priority.

The UK is considered a world leader in palliative and end of life care provision and research, and has influenced end of life care around the world.^2^ However, in the UK and all the more advanced countries, there is evidence of shortfalls, highlighting the challenges facing all countries.^3^ Palliative care is a high priority for the UK National Health Service (NHS) and many other countries; several national guidelines^4^ have been developed over recent years. A review of end of life care in the UK^5^ expressed serious concerns about a lack of research in this field and underuse of existing research. However, research in this area is underfunded compared with studies into the prevention and cure of life-limiting conditions. Less than 0.3% of the £500 million spent on cancer research is allocated to palliative care,^6^ with funding for non-cancer conditions likely to be even less.

Funding leading-edge, needs-led research is essential to improve palliative care across all disease areas. Although the proportion of funding allocated to palliative care research is historically small, funding organisations in the UK, such as the National Institute for Health Research (NIHR) and the partners within the National Cancer Research Institute (NCRI), are helping to address this. Alongside the NCRI, the NIHR is a large funder of palliative care research, also offering resources and support to researchers in the allied fields of dementia and care in the community to the sum of £5.5 m to date.

Support from the NIHR, which funds evidence-based research to support decision-making by clinical teams, patients, carers and policymakers, has advanced palliative care through a growing number of studies of innovative treatments and models of care in the past 10 years. The recent study into patient-reported improvement in breathlessness using an integrated support service is an example of this; it has shown the potential to improve patient quality of life and symptom control with no additional costs to the NHS.^7^ This unique approach gave the first evidence of the benefits of early integration of palliative care for patients with non-cancer conditions and has raised significant interest internationally.

The NIHR funds an array of research programmes evaluating the effectiveness and impact of healthcare treatments and services, supporting researchers from the formation of their research ideas to delivery of evidence-based results to help inform national policies. Importantly, NIHR does not fund in disease siloes. This approach is especially suitable for palliative care, with its emphasis on the patient and family first, rather than their disease, and with a recognition that so many patients experience multimorbidity. All NIHR programmes encourage high quality funding applications that will lead to benefits for patients, carers and the NHS, using either commissioned or researcher-led work streams. More information on funding is available from the NIHR, alongside details of how researchers, clinicians and members of the public can contribute to future research.

In support of the national guidelines’ recommendations to target funding towards palliative care priorities, the NIHR has collaborated with several organisations, including the Motor Neurone Disease Association and Scottish Chief Scientist Office, by co-funding the Palliative and end of life care Priority Setting Partnership (PeolcPSP). The PeolcPSP was initiated by Marie Curie and is overseen by the James Lind Alliance. For the first time in palliative care research, this collaboration enabled more than 1400 patients, carers and healthcare professionals to identify and prioritise gaps in the existing evidence that were most relevant to them. This produced a list of 83 questions, with the priorities being (1) how best to provide care outside of working hours to avoid crises and help people stay in their place of choice, and (2) how access to palliative care services can be improved for everyone irrespective of where they live in the UK.^8^

The role of funding organisations is now to support and develop research evidence to meet the needs identified by patients and all those involved in end of life care. In addition to the PeolcPSP initiative, the NIHR also encourages research suggestions from patients, carers and members of the public, whose insights into conditions and treatments are invaluable in shaping relevant and useful research.

Researchers can take full advantage of the funding and resources available from funding bodies, including charitable, national, international sources, by ensuring that their applications clearly demonstrate how their research will add valuable evidence, in particular testing improved treatment and care for NHS patients. However, other barriers to conducting research in palliative care still exist. Research in this field is challenging, not only because sensitive topics must be discussed, but also because patients may be clinically unstable or have complex symptoms. The recent MORECare project, funded by the EME Programme in collaboration with the Medical Research Council, has produced evidence-based guidance on the best methods for designing and carrying out research in palliative care.^9^ One outcome of this is an e-learning component to support researchers in developing their methodology.

A greater evidence base is also needed to develop good models of practice, particularly in supporting generalists’ work, and in meeting patients’ wishes at the end of their lives. For example, in the first study to explore how health professionals perceived the transition of inpatients to palliative care, Gott *et al*^10^ identified challenges faced by general acute staff in handling the transition. These included difficulties in communicating palliative care needs to the patient, and junior staff having few opportunities to input into transitional care. Such issues need further investigation before palliative care policies can be established in acute care settings. A step forward in assisting with this communication is the development of the psychosocial assessment and communication evaluation (PACE) tool, helping to support information sharing and family perceptions of patients’ symptom control in acute care.^11^ With training in its use from specialist palliative care staff, acute generalists can help improve care of patients and their families.

With respect to issues that are of key importance to patients, Addington-Hall *et al*^12^ reported significant variations in out-of-hours care provision, with services varying between and within primary care organisations. The gaps identified by this research were later echoed by the collaborative PeolcPSP, highlighting the benefit of involving patients early in research planning so models of care can take their needs into account.

Patients with palliative care needs are often admitted to hospital inappropriately when their condition deteriorates. Yet evidence has shown that good access to 24 h community care is likely to reduce the number of emergency hospital admissions.^13^ While many patients express their wishes to die at home, the GUIDE_Care project found that two in five people with dementia die in hospital, although the increasing trend towards hospital deaths was reversed between 2001 and 2010,^14^ largely due to increased care home bed provision. Furthermore, GUIDE_Care's large-scale study investigating place of death over a 27-year period found that nearly two-thirds of 13 million deaths in England occurred in hospital, followed by home or care home, depending on the cause of death.^15^ Funding studies such as these demonstrates how good service provision can help patients stay in their preferred setting, while reducing the strain on overstretched emergency departments and budgets.

Other studies underway with the potential to influence palliative care across all disease areas include an investigation into whether early referral to specialist services produces better outcomes for patients with advanced lung cancer (S Ahmedzai, personal communication, 2015), and development of a support tool to enable patients to manage pain medications in their own home (M Bennett, personal communication, 2015). Once completed, all NIHR research outputs are open access and researchers are encouraged to publish widely. In 2014 the NIHR also commissioned the Cochrane Palliative Care Library to create a searchable database of existing research in the field, covering relevant topics and a range of study designs to inform researchers’ and clinicians’ work around the world.

Research carried out in the UK continues to improve care nationally and through wide dissemination aims to contribute valuable evidence to international palliative care communities. Adopting an international collaborative approach to research is becoming increasingly important to address priorities in end of life care. It is equally important that funding bodies around the world can share lessons learned from successful funding frameworks that support research focused on benefits for patients, their families and those close to them.
